# Patients’ perceptions of podoconiosis causes, prevention and consequences in East and West Gojam, Northern Ethiopia

**DOI:** 10.1186/1471-2458-12-828

**Published:** 2012-09-28

**Authors:** Yordanos B Molla, Sara Tomczyk, Tsige Amberbir, Abreham Tamiru, Gail Davey

**Affiliations:** 1 Brighton and Sussex Medical School, Falmer, Brighton, BN1 9PS, United Kingdom; 2 TOMS, London, United Kingdom; 3 International Orthodox Christian Charities, Addis Ababa, Ethiopia; 4 International Orthodox Christian Charities, Debre Markos, Ethiopia

## Abstract

**Background:**

Podoconiosis is a form of non-filarial elephantiasis that affects barefoot individuals in highland tropical areas. The disease presents with bilateral, asymmetric swelling of the legs, usually confined to below the knee. This study aimed to assess podoconiosis patients’ perceptions of prevention, control, causes and familial clustering of the disease, and to document physical, social and economic impairments associated with the disease, with the ultimate aim of enabling development of tailored interventions in this region.

**Methods:**

This descriptive study is part of the largest cross-sectional community-based household survey yet conducted on podoconiosis. It was completed in November and December, 2011, in Debre Eliyas and Dembecha Woredas of East and West Gojam Zones, northern Ethiopia, and consisted of a house-to-house census by community health workers followed by interviews of identified patients using a structured questionnaire.

**Results:**

In the 17,553 households surveyed, 1,319 patients were identified. More male as compared to female patients were married (84.6% vs. 53.6%, χ^2^ = 157.1, p < 0.0001) while more female as compared to male patients were divorced (22.5% vs. 3.6%, χ^2^ = 102.3, p < 0.0001). Less than half of the study subjects believed podoconiosis could be prevented (37.5%) or controlled (40.4%) and many (41.3%) did not know the cause of podoconiosis. Two-fifths of the study subjects had a relative affected with podoconiosis. Approximately 13% of the respondents had experienced one or more forms of social stigmatization. The coping strategies adopted by patients to mitigate the physical impairments caused by podoconiosis were: working only occasionally (44.9%), avoiding physically demanding tasks (32.4%), working fewer hours (21.9%) or completely stopping work (8%). Most study subjects (96.4%) had noticed a decline in their income following the development of podoconiosis, and 78% said they were poorer than their healthy neighbours.

**Conclusion:**

This study shows that podoconiosis has strong psychosocial, physical and economic impacts on patients in East and West Gojam Zones of northern Ethiopia. Concerns related to familial clustering, poor understanding of the causes and prevention of podoconiosis all add to the physical burden imposed by the disease. Strategies that may ease the impact of podoconiosis include delivery of tailored health education on the causes and prevention of disease, involving patients in intervention activities, and development of alternative income-generating activities for treated patients.

## Background

Podoconiosis is endemic non-filarial elephantiasis of the lower legs resulting from barefoot exposure to red clay soil of volcanic origin. Podoconiosis is prevalent among subsistence barefoot farmers that live and work in these areas [1]. It results in bilateral progressive chronic swelling of the lower legs, usually limited below the level of the knees. The pathogenesis of the disease has not yet been investigated in depth, but it is believed to be caused by fine particles in the soil that penetrate the skin and induce an inflammatory reaction in the lymphatic system [2]. Podoconiosis is classified into five stages where the first stage swelling is limited to below the ankle and is reversible overnight. The second stage swelling is not reversible, and when bumps and knobs are present they remain below the level of the ankle. In the third stage of the disease, bumps and knobs are found above the level of the ankle. The fourth stage entails above knee swelling whereas the fifth stage involves joint fixation as a result of surrounding soft tissue overgrowth [3]. Podoconiosis can be prevented, early forms of the disease can be treated, and disease progression can be controlled with simple but effective measures such as washing feet with soap and water on a regular basis and wearing protective shoes consistently [4]. Podoconiosis has recently been included in the World Health Organization’s Neglected Tropical Diseases (NTDs) list [5].

One million cases are estimated to exist in Ethiopia, and 64% of these are in the economically productive age group. The average prevalence of the disease in Ethiopia is greater than 5% in endemic areas [1]. In Wolaita Zone of southern Ethiopia, one study showed that the disease results in an annual economic loss of 16 million USD [6]. Through direct projection from these 2005 figures, the economic loss in Ethiopia due to podoconiosis is estimated to be more than 200 million USD per year [6,7].

A disease is deemed to be of public health importance on the basis of either high mortality or morbidity. The disease burden imposed by non-fatal causes of disease affects low and middle income countries more than developed ones. This adds poor quality of life to the short life span of the population in the developing world [8]. Podoconiosis is one such non-fatal diseases, resulting in pronounced disfigurement and debilitation among affected individuals [9]. In podoconiosis-endemic areas the disease is more prevalent than fatal diseases such as HIV/AIDS [10]. Nevertheless, lack of knowledge about the cause of the disease and unfavourable attitudes of community members and health professionals towards patients intensify the burden of podoconiosis [11,12]. Manifestations of stigma linked to podoconiosis include school dropout, lack of marriage prospects, exclusion from community events, and psychological trauma [11,13]. There are no recent accounts of the psychosocial and socio-economic consequences of podoconiosis in Amhara Region, northern Ethiopia although such consequences have been documented in previous studies in southern Ethiopia and north-west Cameroon [11-14]. This study aimed to assess perceptions of podoconiosis patients surrounding prevention, control, causes and familial clustering of the disease and to document physical, social and economic impairments associated with the disease in East and West Gojam Zones of Amhara region. Ultimately, we anticipate that the results of this study will enable the development of tailored intervention strategies in the region.

## Methods

### Ethics statement

The Amhara Regional Health Bureau provided ethical clearance for the study. Support letters were also obtained from East and West Gojam Zonal Health Departments and *Woreda* (District) Health Offices. Since most of the respondents were not able to read and write, the interviewers read the written consent form to them and oral informed consent was obtained from each study participant. This use of verbal consent was approved by the regional ethics review board of the Amhara Regional Health Bureau. Interviewers confirmed the participant’s oral consent by signing the consent form for each interview as per the guideline of the regional ethics review board. Consent was obtained from parents or guardians for children aged less than 18 years (the legal age for giving consent for research in Ethiopia).

### Study design and study area

We did a cross-sectional community-based household survey to explore the burden of podoconiosis in Debre Eliyas and Dembecha Woredas of East and West Gojam Zones of Amhara Regional State. These two Zones are found adjacent to Debre Markos town, which is located 305 Km north from the capital city of Addis Ababa. The population of Dembecha and Debre Eliyas Woreda is 111,317 and 82,150 respectively. The majority of the population are farmer and Amharic language speakers in both Woredas [15]. Villages (*ketena*) in known podoconiosis-endemic *kebeles* (the lowest level government administrative structure in Ethiopia) were included in the house-to-house case enumeration. Identification of the study area was based on a report by the International Orthodox Christian Charities (IOCC) podoconiosis treatment center, written in 2010, summarizing information from key local informants. The study participants were residents of the selected *kebeles* and podoconiosis cases in all households with podoconiosis.

### Sampling procedure and sample size determination

The Ethiopian administrative structure is organized hierarchically, with multiple Zones in each Region. Each Zone contains multiple *woredas* (equivalent to districts). Each *woreda* contains *kebeles* and each *kebele* contains villages with multiple households. A convenience non-random sampling method was used to select two Zones. A list of *woredas* in East and West Gojam Zones, known for the presence of podoconiosis based on expert opinion and key informants, was prepared. Next, a random sampling technique was applied to select two *woredas* from this list, one from each Zone. Finally, *kebeles* from each of these two *woredas* were randomly selected. The number of *kebeles* selected was proportional to the population size of each *woreda*. A total of 20 *kebeles* from two *woredas* in East Gojam and West Gojam Zones (7 *kebeles* from East Gojam and 13 *kebeles* from West Gojam) were selected.

All households in the selected *kebeles* were assessed for the presence of podoconiosis cases through interviews with the household head followed by clinical examination of cases by community health extension workers (HEW). Podoconiosis cases were invited to participate in further interviews and examinations with clinical nurses. In households where there was more than one podoconiosis patient, all patients were invited to interview and physical examination.

### Data collection process

Data collection was done by trained HEWs supervised by clinical nurses working in the respective *woredas*. The HEWs were responsible for house-to-house enumeration of podoconiosis cases and the nurses were responsible for supervising the activities of the HEWs and the detailed assessment of podoconiosis cases (i.e., interviewing and physical examination of patients). Before performing data collection, all HEWs and nurses received training from the research coordinators on: techniques and approaches for obtaining informed consent from prospective participants, interviewing techniques, podoconiosis diagnostic features, clinical staging according to a standard method, assessment of acute adenolymphangitis (ALA, a common sequela of lymphoedema), measurement of leg circumference (the largest circumference between the levels of the ankle and knee measured using a tape, to a precision level of the nearest centimeter), assessment of presence of open wounds, and features that differentiate lymphoedema and leg swelling resulting from podoconiosis from other diseases such as leprosy and filarial elephantiasis. They were also trained to advise patients to wear shoes and wash their feet to control disease progression at the end of every interview. The data collection process was supervised by the study investigators.

A pre-test was conducted immediately after the training of the data collectors. The pre-test was done in two *kebeles* (one in West Gojam *Woreda* and the other in East Gojam *Woreda*) which were not included in the main survey. The pre-test was evaluated in terms of (i) organization of the fieldwork and coordination between the team of investigators, nurses and HEWs; (ii) ability of the HEWs to effectively conduct the census and complete the questionnaires; (iii) ability of the nurses to correctly diagnose and stage podoconiosis, and identify ALA symptoms (painful inflammation of the foot and leg with swollen lymph nodes and fever); (iv) completeness, skip patterns, flow and clarity of the questionnaire. At the end of the pre-test, the trainees brought back the data they collected to the training centre, where questionnaires were checked by the trainers. Discussion was held on the challenges the HEWs faced during data collection and regarding the data collection tools which led to the revision of the questionnaires.

### Data collection tools

The data collection tool was a structured questionnaire. The questionnaire was developed in English, translated into Amharic and back translated into English to check consistency. The questionnaire was sub-sectioned thematically to include: socio-demographic characteristics; podoconiosis and ALA history; knowledge about podoconiosis causes, prevention, control and treatment; stigma experience; clinical features; treatment-seeking behaviour; family history of podoconiosis; walking practices; foot hygiene; water sources; shoe wearing practices and podoconiosis related impairments.

### Data processing and analysis

Data were entered and analysed using the Statistical Package for Social Sciences (SPSS) software v.17.0. Statistical significance was tested using the chi-squared test or t-test as appropriate to determine if socio-demographic characteristics such as sex were associated with resulting social stigma or disease occurrence and if there were differences between the average values observed in different groups such as age. The level of significance was set at α of 0.05.

## Results

### Socio-demographic characteristics of study participants

The socio-demographic characteristics of the study participants have been described in an earlier article [16]. In brief, from 17,553 houses, a total of 1,319 podoconiosis cases were identified and interviewed. Slightly over half (50.8%) of these cases were men, most were adults in the productive age group (15–64 years, 88.9%), who did not read or write (79.3%), and worked as farmers (74.5%). On average, patients had lived in the study area for 40 years. More male than female patients were married (84.6% vs. 53.6%, χ^2^ = 157.1, p < 0.0001) and divorce was more common among women than men (22.5% vs. 3.6%, χ^2^ = 102.3, p < 0.0001).

### Perceptions about the cause, prevention and control of podoconiosis

In the interviews, patients were asked what they thought caused podoconiosis. Many (41.3%) said they did not know the cause, while others conjectured barefoot walking (18%), heritability (7%), and exposure of feet to condensation (7.4%). Additional responses included a curse from God or the action of a witch, injury and *mitch* (exposure to sunshine resulting in inflammation).

Patients were next asked whether they thought podoconiosis could be prevented and controlled. More than one third (37.5%) believed podoconiosis to be a preventable disease and 40.4% believed that disease progression could be controlled. The remaining respondents said they either ‘knew’ or ‘thought that’ podoconiosis could not be prevented (22.2% and 40.3%, respectively) or controlled (27.3% and 32.3%, respectively). Patients who believed that podoconiosis could be prevented mentioned the following methods of prevention: wearing shoes (82.1%), washing feet (19.1%), avoiding contact with an affected person (3.6%), and avoiding marriage to podoconiosis patients (1.3%). Medical intervention and *tsebel* (holy water) were mentioned by very few. Study participants who believed that progression of podoconiosis could be controlled mentioned the following possible methods: wearing shoes (87.0%), washing feet (17.6%), medical treatment (55.4%), and *tsebel* (35.7%). The reasons given by the study participants who thought that podoconiosis could not be controlled or cured were: absence of drugs for the disease (54%), having not seen a cured patient (51.9%), and because podoconiosis is hereditary (8.7%).

### Perceptions about familial clustering of podoconiosis

**Figure 1:**
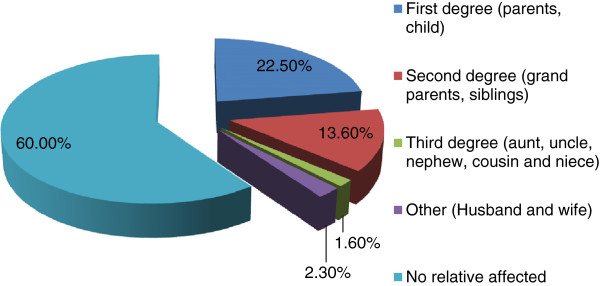
**Familial clustering of podoconiosis,East and West GojamZones, northern Ethiopia**.

Clustering of podoconiosis patients within a family was observed. A total of 40% of participants said that there were other patients in their family, of whom most were first (22.5%) or second (13.6%) degree relatives (Figure 1). When asked why there might be multiple affected members of one family, most (45.8%) said they did not know, 21% said because podoconiosis is contagious, 18.4% that it is hereditary and 9.6% that it is a curse on the family from God.

### Social stigma

Approximately 13% of patients mentioned that they had experienced one or more forms of social stigmatization at school, church, or in the market place. The various forms of stigmatization and the locations at which these were experienced are described in Table 1. There was a statistically significant difference between men and women in exclusion from marriage and shunning within marriage (0.8% % vs. 3.6%, X^2^ = 12.7, p < 0.0001), but there was no statistically significant difference between men and women for any other experience of stigma.

**Table 1:** **Types of stigma experiencedby study subjects inEast and West GojamZones, northern Ethiopia**

**Type of stigma (n** **= 1303)**	**Places**					
	**School**	**Church**	**Marriage**	**Market**	**Feast/ Holiday gathering**	**Non-specific**
**School drop-out**	17 (1.3%)					
**Forced exclusion**		25 (1.9%)	22 (1.7%)		40 (3.1%)	
**Not buying their products**				26 (2%)		
**Shunning**	14 (1.1%)	33 (2.5%)	18 (1.4%)		29 (2.2%)	
**Pointing at them**	5 (0.4%)	25 (1.9%)	2 (0.2%)		10 (0.8%)	
**Pinching nose**	3 (0.2%)	19 (1.5%)	0 (0%)		13 (1%)	
**Insulting**						19 (1.5%)
**Total**	**39 (3.0%)**	**102 (7.8%)**	**42 (3.2%)**	**26 (2.0%)**	**92 (7.1%)**	**19 (1.5%)**

### Physical and productivity impairments

We assessed patient perception of the effect of podoconiosis on physical activity and economic productivity. Many (60.1%) said that their movement was impaired by podoconiosis and 26.9% said their movement was frequently impaired by episodes of ALA . More than half (55.0%) of women claimed their household chores were affected by podoconiosis, and 30.9% that chores were affected by ALA. Similar proportions of men said their daily activities were impaired by podoconiosis (59.5%) and ALA (23.6%).

Many participants (36.8%) believed that podoconiosis reduced the energy with which they could work, 38.2% stated that it reduced their working hours, and 35.0% stated that it increased work absenteeism. Several patients thought that their productivity had declined (24.6%), and 16.6% believed that this meant they could not earn as much income as podoconiosis-free individuals in their community. The coping strategies adopted by patients to mitigate the physical impairment caused by podoconiosis included: working only occasionally (44.9%), avoiding physically demanding tasks (32.4%), working shorter hours (21.9%) or completely stopping work (8%). Almost all (96.4%) patients said they had experienced a decline in their income following the development of podoconiosis. When asked to rate their income compared to their healthy neighbours, 78% said they were poorer.

## Discussion

This descriptive study is part of a large cross-sectional community-based household survey that was conducted to explore the impact of podoconiosis in northern Ethiopia. In this report, we document extensive effects of podoconiosis-related psychological, physical and economic impairments on patients. We also confirm the existence of substantial misconceptions surrounding causes, prevention, control and the familial nature of podoconiosis.

Approximately half of the patients interviewed had misconceptions about the cause of podoconiosis. Less than half of patients believed podoconiosis could be prevented (37.5%) or controlled (40.4%). Among individuals who said that podoconiosis could be prevented, some believed that avoiding contact with patients through physical contact might be helpful. Some suggested that control might be possible by avoiding contact with patients (assuming podoconiosis was infectious) or by using holy water (assuming the cause was supernatural). Avoidance of contact may partly explain the high divorce rates among our study subjects. Previous studies in the southern and northern parts of Ethiopia and north-western Cameroon have also observed multiple misconceptions about the cause of podoconiosis including witchcraft or contact with podoconiosis patients among the community [11,14,17]. These misconceptions have an effect on the individual, the community and on podoconiosis programs and interventions. Like other debilitating diseases such as leprosy and lymphatic filariasis, patients with podoconiosis may find it difficult to be accepted by communities because of exclusion from community events and marriage [18,19]. The belief that podoconiosis is contagious could make communities avoid contact with patients and health professionals be unwilling to provide health care [11,12]. Myths about the causes, prevention and treatment of podoconiosis may mean that patients look for alternative ways to treat the disease, avoid seeking treatment, or be less likely to adopt shoe wearing and foot hygiene [17]. Intervention must target these knowledge gaps through education directed not only to people with podoconiosis but also to the general community. Involving patients that have witnessed improvement through adherence to treatment, as experienced by an intervention program in the southern part of Ethiopia needs to be scaled up to other endemic areas [20]. Podoconiosis care and support must also be integrated with national neglected tropical disease initiatives such as those focusing on the importance of hygiene.

Approximately two-fifths of participants had a family member affected with podoconiosis. We found that more patients stated that they had an affected first degree (parent or child) or second degree (grandparent or siblings) relative than a more distant affected relative. However, this may be affected by recall bias or because of loose family ties beyond first and second degree relatives. Participants assumed that podoconiosis clustering within families meant that it could not be prevented or controlled. Previous studies in southern Ethiopia have shown widespread beliefs about the familial nature of podoconiosis [11,21]. Segregation analysis [22] and more recently a genome-wide association study [23] have demonstrated genetic susceptibility to podoconiosis. A recent study in southern Ethiopia has explored the influence of beliefs about heritability on behaviour, and suggests that those who think podoconiosis is heritable are less likely to endorse prevention of disease by use of shoes [24]. Similar consequences of beliefs about heritability are seen in the present study, with 8.7% of respondents giving this as a reason for thinking that podoconiosis could not be prevented or treated. Participants in another study in southern Ethiopia used more biologically-based terms such as ‘bone’, and ‘blood’ [25] to describe familial clustering than did our present study participants. A more detailed study is needed to understand better the extent of existing understanding about familial clustering and stigma in north Ethiopia. Even though familial clustering of podoconiosis is a source of stigma against patients, the benefits of targeting and prioritizing high risk groups for intervention should be taken into account [26].

Observing that podoconiosis clusters in families, and believing that it cannot be prevented or treated are important sources for social and psychological distress among affected people. Intense stigmatization of podoconiosis patients has been shown to be widespread by previous studies [11-14,25,27]. In this study, podoconiosis patients experience “enacted” stigma in the form of isolation from community events such as feasts and church ceremonies. This may lead to “internalising” (accepting) the stigma [28]. Tora *et al.* explored the psychological effect of stigma, documenting how stigmatised patients opt for avoidant types of coping which in turn separates patients further from non-patients and aggravates stigma-induced stress [13]. These observations demonstrate the need for further investigation to measure the extent of psychological trauma and the mental health status of podoconiosis patients. In addition to exacerbating emotional distress, stigma may delay treatment seeking and affect adherence to treatment as seen in this study and previous research [28,29].

This study is broadly consistent with others in southern and south-western Ethiopia and north-western Cameroon that have documented the social andeconomic impact of podoconiosis in endemic areas [14,25,30]. Functional impairments such as inability to move or to do daily occupational work or household chores hamper patients’ physical capabilities. As with other debilitating diseases such as lymphatic filariasis, functional impairment worsens the burden of the disease [31] and can affect economic productivity of patients. Patients reported that they worked fewer hours, stopped work or were only able to work with reduced energy. They also perceived their productivity and income to have decreased more since becoming affected with podoconiosis than their healthy neighbours. Similarly, experiences from families of lymphatic filariasis patients show that the economic loss due to the disease may push an already poor family to near destitution [29]. The interconnection of lymphatic filariasis with poverty acts beyond the household level, and contributes to poverty at national level, reducing the likelihood of achieving the Millennium Development Goal related to poverty reduction [32,33]. Likewise, economic analysis of productivity loss due to podoconiosis indicates enormous economic losses in southern Ethiopia [6], and the subjective reports of this study suggest that similar losses occur in other podoconiosis-endemic areas of Ethiopia. This productivity loss due to podoconiosis cannot be addressed with mere treatment of the disease. Alternative income generating activities that are less physically demanding are needed.

This study has a number of limitations. Any study of a disease as stigmatised as podoconiosis is likely to face issues of non-response bias: families may deny the presence of affected members or even actively hide them, resulting in under-counting of podoconiosis cases. We attempted to minimize under-counting by using HEWs as data collectors. HEWs are familiar, trusted, community members, who know most families in their *kebeles*. However, it is still possible that some families concealed members with disease. Another limitation was that we assessed economic losses only through relatively crude patient reports. No form of quantitative measurement of income, expenditure or productivity was made in patients or community controls. Similarly, stigma assessment was based purely on patient report: we did not use a standardised tool or collect information on stigma from the wider community. We believe that this survey still valuably documents the existence of social and economic consequences of podoconiosis, even though we do not claim to accurately quantify these consequences.

## Conclusion

Overall, this study shows that in North Ethiopia, podoconiosis has strong psychosocial, physical and economic impacts on the lives of patients. Concerns related to familial clustering, and poor understanding of the causes, prevention and control of the disease add to the disease burden. The high prevalence and profound consequences of podoconiosis in this area call for health education about the causes, prevention and control of podoconiosis to be integrated into national Neglected Tropical Disease initiatives. The extent of psychological distress due to stigma needs further exploration and carefully tailored interventions. The familial nature of the disease may enable targeted intervention, so families of the affected are given priority in resource-constrained areas [26]. The economic disadvantages that result from physical impairment due to podoconiosis may be averted by introducing alternative income generating activities.

## Competing interests

The authors declare that they have no competing interest.

## Authors’ contributions

YBM, ST, TA, AT and GD designed the study. YBM, ST, TA, AT did the fieldwork. YBM analyzed the data and drafted the manuscript. All authors revised the paper for substantial intellectual content. All authors read and approved the final manuscript.

## Pre-publication history

The pre-publication history for this paper can be accessed here:

http://www.biomedcentral.com/1471-2458/12/828/prepub
